# Effects of Ecological Complexity on Student Identification Accuracy in a School-Based Citizen Science Program

**DOI:** 10.3390/biology15100787

**Published:** 2026-05-15

**Authors:** Motti Charter

**Affiliations:** 1 Shamir Research Institute, University of Haifa, Katzrin 1290000, Israel; mcharter@geo.haifa.ac.il; Tel.: +972-544901130; 2 School of Environmental Sciences, Faculty of Social Sciences, University of Haifa, Mount Carmel, Haifa 3498838, Israel

**Keywords:** citizen science, barn owl pellets, biodiversity monitoring, identification accuracy, ecological complexity, observer error

## Abstract

School-based citizen science is increasingly used to support biodiversity monitoring, but the reliability of student-generated ecological data remains uncertain. I evaluated a nationwide barn owl (*Tyto alba*) pellet project in Israel involving 1612 students and 107 teachers over two academic years. Teacher participation was moderate to high: 66.4% of teachers returned at least one complete kit, generating 3333 prey records, mostly from small mammals, thereby demonstrating that schools can contribute significant ecological sampling effort across broad geographic areas. Student identification accuracy was moderate: 50% of pellet analyses were perfectly identified, with an overall item-level accuracy of 62%, and 76% of pellet attempts included at least one correctly identified prey item. The probability of perfect identification declined sharply with pellet complexity. Identification accuracy also varied among prey taxa: even under minimal complexity (single-species pellets), certain taxa exhibited significantly higher misidentification rates than others. Each additional prey item reduced the odds of correctly identifying all prey items in a pellet by about 68%, indicating that identification reliability decreases as sample complexity increases. Although many students initially found pellets disgusting, engagement remained high, and willingness to repeat the activity was strongly linked to enjoyment. These findings show that school-based citizen science can support biodiversity monitoring, but reliable ecological inference requires validation frameworks, especially when sample complexity is high.

## Simple Summary

School-based citizen science can help gather large amounts of information about nature, but the quality of student-created data is often uncertain. I examined a nationwide barn owl pellet project in Israel where students analyzed owl pellets to identify prey remains. My aim was to determine whether schools can provide useful biodiversity data and how task difficulty impacts accuracy. I found that many teachers successfully completed the project and submitted usable samples, demonstrating that schools can contribute significant ecological data across a broad region. Student accuracy in identifying prey was moderate: many students correctly identified some prey items, but accuracy dropped sharply when pellets contained more prey remains. This indicates that as natural samples grow more complex, accurate identification becomes more difficult. Although many students initially found pellets unappealing, most remained engaged, and many expressed a willingness to repeat the activity. My results show that school-based citizen science can support biodiversity monitoring, but projects involving detailed species identification should incorporate expert review or other validation methods to ensure dependable results.

## 1. Introduction

Scaling biodiversity monitoring over large areas is a key challenge in applied ecology [1,2]. Citizen science has emerged as a key approach to addressing this challenge by involving human participants directly in ecological data collection [3]. In human–natural systems, ecological knowledge is created through interactions between observers and the environment [4,5]. Reliable monitoring of small mammal communities is particularly important for understanding biodiversity dynamics [6], ecosystem services [7,8], and agricultural pest regulation [9]. However, conventional trapping approaches are labor-intensive, costly, and spatially constrained, prompting the use of indirect dietary methods such as owl pellet analysis [10].

Owl pellets are also used to determine the composition of small mammal communities and can provide a better representation of these communities compared to estimates from conventional trapping [11,12]. However, debate persists over how accurately pellet contents reflect prey availability in the field. Some studies suggest that pellet contents approximate local prey assemblages under certain conditions [13], whereas others demonstrate biases related to prey abundance, habitat use, or predator selectivity [14], 2016). Differential predation across prey size classes and sex has also been documented [15], and species-level prey selection patterns may vary regionally [16]. These considerations highlight that pellet-derived data reflect both prey availability and predator behavior.

Barn owls (*Tyto alba*) are one of the most common small mammal specialists [17], whose diet can fluctuate with changes in prey abundance, and are found throughout the world. Since barn owl pellets are easy to collect, store, and examine [18], diet studies based on them also serve as practical platforms for public participation in ecological research. However, when non-expert observers collect ecological data, the reliability of their inferences depends not only on their training and expertise but also on how human perceptual and cognitive constraints interact with ecological complexity [19,20]. Ecological inference from dietary items often requires reliable estimation of species composition, proportional abundance, and detection of rare taxa. Even moderate identification error may bias these estimates, particularly in samples containing multiple prey species. In Israel, barn owls are widely deployed as biological control agents in agriculture [21,22] to reduce rodent damage, resulting in extensive nest-box networks [23] and long-term dietary monitoring [24,25] across the country.

Citizen science has grown rapidly as a method for expanding biodiversity monitoring, biological research, and science education [26], but concerns remain regarding data accuracy, with quantitative evaluations showing that volunteer-generated data are sometimes, but not consistently, comparable to professional standards [27]. Empirical evaluations show mixed results: in some cases, student measurements approximate professional data, but greater variance raises concerns about precision for ecological monitoring [28], while detailed error analyses reveal frequent procedural and identification mistakes [29]. Most evaluations implicitly treat observer error as random variation or as a result of training quality. However, less attention has been paid to whether identification error arises predictably from interactions between observer capacity and the structure of ecological samples [30,31]. Within education, science education experts primarily focus on children’s learning experiences [32,33,34], and science educators often justify implementing citizen science in schools by citing data-quality evidence from adult-based citizen science projects [35].

Few studies have rigorously evaluated whether these programs generate data reliable enough for species-level ecological inference under authentic classroom conditions [3,36]. Even in studies reporting acceptable average accuracy, it remains unclear whether identification errors systematically increase with task complexity. This uncertainty limits the ecological conclusions that can be drawn from student-generated data. These concerns extend beyond pellet analysis to any school-based activity requiring morphological or taxonomic discrimination, as similar variation in volunteer-generated data quality has been documented across citizen science contexts [37]. Therefore, distinguishing between correctable training deficiencies and performance limits caused by inherent complexity is essential for developing scalable ecological monitoring frameworks.

Teachers act as crucial mediators in shaping how ecological data are collected, as observer traits and behavior directly influence data quality and interpretation [19,26,38]. In school-based citizen science programs, an additional concern is whether teachers can reliably follow standardized protocols and support the submission of scientifically valid data without direct supervision. Emerging research indicates that teachers’ ability to effectively facilitate school-based citizen science may be limited without structured professional development and ongoing support [39]. Teachers often report limited methodological guidance, challenges in choosing appropriate tools, and difficulties designing scientifically rigorous activities, showing that implementation fidelity cannot be assumed [40]. Additionally, case study research reveals significant variation in how teachers implement citizen science curricula, even with structured support materials, highlighting the influence of contextual and instructional factors on data quality in classroom settings [41].

The validity of biodiversity monitoring depends on the relationship between observation error and ecological complexity. When multiple taxa co-occur within samples, misclassification does not simply reduce accuracy; it can systematically distort community metrics and obscure rare species [19,42]. Understanding whether identification error scales predictably with ecological complexity is therefore central to determining whether citizen science systems can generate reliable ecological knowledge [37,43]. However, this relationship has rarely been quantified in school-based monitoring systems.

In community monitoring, detection probability and misclassification error jointly determine whether species are recorded or overlooked [44]. When identification error increases with sample complexity, apparent absences may reflect observer limitations rather than ecological rarity, thereby biasing inferences about species richness and community composition. If identification error increases systematically with ecological complexity, then biases in species detection and abundance estimates may arise as inherent properties of the interaction between observers and ecological systems, rather than as correctable noise [19,30].

Although citizen science research has emphasized training and validation, little attention has been given to whether intrinsic ecological complexity systematically constrains identification accuracy in school-based programs. It remains unclear whether error rates increase predictably with sample complexity, thereby limiting ecological inference from student-generated data.

This study assessed a nationwide school-based citizen science program to see how ecological complexity affects student identification accuracy in real classroom settings. Specifically, I examined: (1) whether teachers are likely to complete and return standardized research kits; (2) if student prey identifications are accurate enough to support ecological diet analysis; and (3) whether increasing task complexity reduces identification success. This framework allows us to assess not only whether students participate successfully, but also how ecological complexity affects the reliability of student-generated ecological data. By analyzing how identification errors vary with pellet complexity, I determine when school-based participation can support species-level ecological inference and when expert validation becomes necessary.

## 2. Materials and Methods

### 2.1. Study Design

I implemented a nationwide school-based citizen science program in Israel during the 2018–2019 and 2019–2020 academic years to evaluate teacher participation, student engagement, and student accuracy in prey identification from barn owl pellets. The project was embedded within ongoing ecological monitoring of barn owl diet and designed to assess both feasibility and data reliability under routine classroom conditions. A total of 107 teachers from 74 middle and high schools received standardized pellet analysis kits after participating in professional development workshops. Classroom implementation occurred without the researcher’s presence. Across both years, 1612 students conducted pellet dissections, and 412 completed post-activity questionnaires assessing perceptions and engagement. Participating schools were recruited through the Ministry of Education networks following the teacher training workshops. Schools were not randomly selected; participation was voluntary and limited to teachers who attended the workshops and elected to implement the program in their classrooms. Thus, the sample represents self-selected middle and high schools that opted to participate under standard classroom conditions.

### 2.2. Teacher Recruitment and Training

Teachers were recruited through official workshops of the Israel Ministry of Education, which invited the study author to deliver pellet-analysis training as part of its professional development program. The relevant Ministry supervisors were therefore familiar with the workshop content, materials, and implementation procedures used in the project. All workshops were designed and delivered by the study author (MC), a university lecturer and researcher specializing in barn owl ecology and trophic dynamics. Participating teachers attended six-hour professional development workshops. Training included instruction on barn owl ecology, study objectives, standardized pellet dissection procedures, and prey identification using skeletal morphology and dentition keys.

Teachers independently dissected pellets during the workshops to ensure full familiarity with the protocol and the ability to implement the activity without researcher supervision. Because the workshops were conducted by an active researcher whose ecological monitoring relies on pellet analysis, teachers received direct training from a subject matter expert using the same identification framework employed in ongoing academic research. In addition to pellet identification procedures, the workshops introduced teachers to basic data compilation and analysis in Excel, including summarizing prey frequencies and generating simple descriptive statistics and graphs. Teachers were encouraged to have students conduct small classroom-level analyses of their pellet data following dissection. However, student data analysis performance was not formally measured or evaluated in the present study. Participation in the classroom phase was voluntary following training.

### 2.3. Research Kits and Materials

Each participating teacher received individually numbered research kits designed to standardize sampling and data recording. Kits contained 24 sterilized barn owl pellets packaged into two geographic subsets, a magnifying glass, written implementation protocols, 24 uniquely labeled specimen bags for skeletal items, prey identification keys, student questionnaires (both academic years), a teacher questionnaire (primarily 2019–2020), and prepaid return shipping materials. An Excel template for summarizing pellet data was included in the instructional materials. The pellets were collected from agricultural landscapes in northern Israel, as described in [24], where barn owls are widely used for biological rodent control.

Each pellet was assigned a unique identifier linking the student-recorded identifications to the returned skeletal items. Students were instructed to isolate and preserve all identifiable skeletal elements from each pellet and to record prey determinations on standardized data sheets. The owl pellets were sterilized in a hot-air oven at 160 °C (320 °F) for 2 h to kill potential pathogens.

Owl pellet analysis is a well-established method for reconstructing small mammal communities and trophic interactions in agricultural systems [13,14]. Long-term monitoring of pellets at these sites indicates that barn owl pellets from the study region typically contain remains of approximately 5–8 small mammal taxa, primarily *Microtus guentheri*, *Mus musculus*, *Meriones tristrami*, *Rattus rattus*, and *Crocidura* spp. [24,25,45]. Species richness within individual pellets varies substantially, with some pellets containing a single prey item and others containing multiple individuals from different taxa. This natural variation in prey composition provided the basis for evaluating how ecological complexity influences identification accuracy.

In addition to the identification keys, teachers received a detailed skeletal reference sheet illustrating three common rodent species, with all major cranial and mandibular elements labeled in Arabic, Hebrew, and English. The sheet was designed to standardize anatomical terminology across schools serving linguistically diverse student populations. Teachers were instructed to print and distribute the reference sheet during dissection sessions. Each kit also included printed identification guides focused on the most common rodent taxa expected in barn owl pellets from the study region, emphasizing diagnostic dental and cranial characteristics relevant for species-level identification. The identification framework followed established osteological criteria for small mammal skeletal and dental morphology commonly used in barn owl pellet analyses in the region [18,24,45]. The skeletal morphology and dentition identification keys used in the teacher training workshops are publicly available at: [https://shorturl.at/6GJmW (accessed on 12 May 2026)].

### 2.4. Classroom Implementation

Prior to dissection, students received approximately 1 h of structured instruction using standardized project materials, including presentation slides, instructional videos, and identification keys prepared by the study author. During dissection, pellets were hydrated and manually separated into fur and skeletal components. Students isolated skulls and mandibles and identified prey taxa primarily based on dental characteristics. All identifications were conducted within the classroom under teacher supervision. Researchers were not present during classroom implementation. Following dissection, teachers were encouraged to guide students in compiling class-level prey data and conducting simple descriptive analyses in Excel; however, the extent and quality of this component were not systematically recorded and are therefore not included in the present analyses. After completing the activity, students completed questionnaires assessing disgust toward pellets, enjoyment of the activity (1 = suffered to 5 = enjoyed a lot), and willingness to repeat the activity (1 = no to 5 = definitely). Teachers completed a separate questionnaire regarding prior experience with classroom research and perceptions of the project.

### 2.5. Expert Verification and Identification Accuracy

All skeletal items and identification forms were returned to the researcher. All returned items were examined and verified by the study author, who routinely conducts professional pellet-based dietary analyses as part of long-term barn owl monitoring programs in Israel (e.g., [24,25,45,46]). Expert determinations were treated as the reference standard for prey identity. Student identifications were compared directly with expert determinations at the level of individual prey items within each pellet.

Prey identification performance was quantified at the student–pellet level using three complementary metrics: (1) perfect identification (binary; all prey items correctly identified), (2) proportional accuracy (number of correctly identified prey items divided by total prey items within the pellet), and (3) any success (binary; at least one prey item correctly identified). Similar accuracy metrics have been applied in prior citizen science validation studies to evaluate identification reliability [37]. Pellet complexity was defined as the total number of prey items contained within each pellet and was treated as a continuous predictor of identification difficulty. In this study, ecological complexity is operationalized as the total number of distinct prey remains within a single pellet. Pellets containing more prey remains require greater taxonomic discrimination and increase the potential for misclassification, as students must identify multiple skeletal elements that may belong to different species. Thus, ecological complexity refers specifically to structural properties of the biological sample rather than to habitat-level or community-level ecological metrics.

Similar multi-level accuracy metrics, including item-level accuracy, complete identification success, and binary correct/incorrect classifications, have been used in prior citizen science validation studies to evaluate identification reliability and observer performance [19,37,47]. These approaches allow assessment of both overall proportional accuracy and threshold-based identification success.

### 2.6. Response Variables and Covariates

Teacher participation was quantified as a binary outcome (returned at least one completed kit) and, among returning teachers, as the proportion of completed kits relative to the number of kits taken. Teacher background variables, available only for returning teachers who completed the teacher questionnaire, included prior experience conducting classroom-based scientific activities and prior collaboration with academic researchers (both binary). Student-level covariates included grade level (middle vs. high school), gender, school geographic region (central vs. peripheral), perceived disgust (binary), enjoyment (1–5 Likert scale), and willingness to repeat the activity (1–5 Likert scale).

Participation by teachers and students was voluntary, and student questionnaires were completed anonymously. No names or identifying personal information were collected from students. The project was conducted under the auspices of the Israel Ministry of Education and implemented through official Ministry workshops, with the knowledge and support of the Israeli Supervisor of Biology, Science, and Mathematics for High Schools (Dr. Irit Sadeh) and the Science and Technology Supervisor for Middle Schools in the Northern District (Mrs. Nirit Berger). Because the activity was implemented as an official Ministry of Education school-based project, separate written parental consent procedures were not required under institutional and Ministry of Education guidelines.

### 2.7. Statistical Analyses

Local prey-identification difficulty (“pellet complexity”) was quantified as the total number of prey items in each pellet. For each student–pellet attempt, prey identification performance was summarized in three complementary ways: (1) overall accuracy, defined as the number of prey items correctly identified out of the total number of prey items in that pellet; (2) perfect identification, coded as whether all prey items in the pellet were identified correctly (0/1); and (3) any success, coded as whether the student identified at least one prey item correctly (0/1). Student perceptions were summarized using (i) whether pellets were perceived as disgusting (yes/no), (ii) enjoyment of the activity (1 = suffered to 5 = enjoyed a lot), and (iii) willingness to repeat the activity (1 = no to 5 = definitely). Sample sizes varied among models because analyses were restricted to records with complete responses for the relevant variables.

To evaluate taxon-specific identification difficulty independent of pellet complexity, analyses were restricted to pellets containing a single prey species. This restriction allowed unambiguous assignment of identification success to a specific taxon. Species identity (vole, mouse, jird, rat, shrew) was included as a categorical fixed effect in a binomial generalized linear mixed model with a logit link, with correct identification (0 = incorrect, 1 = correct) as the response variable and school included as a random intercept. Teacher participation was analyzed in three steps. First, among all teachers who received kits, I modeled the probability of returning at least one completed kit using binary logistic regression with school level, school location, and year as fixed effects. Second, among teachers who returned at least one completed kit, I modeled kit completion rate (number of completed kits returned/total kits taken) using a binomial generalized linear model with a logit link, with school level, school location, and year as fixed effects. Third, among the subset of returning teachers who completed the teacher questionnaire, I modeled kit completion rate using a binomial generalized linear model with a logit link, including year, prior classroom scientific activity, and prior collaboration with academic researchers as fixed effects.

To evaluate predictors of student perceptions and prey identification success, I used generalized linear mixed models (GLMMs) [48]. Binary outcomes were analyzed with a binary logistic regression GLMM with a logit link. Overall accuracy was summarized descriptively as the proportion of prey items correctly identified within each pellet. Enjoyment and willingness ratings were analyzed using normal mixed effects models with an identity link (treating 1–5 ratings as approximately continuous) [49]. Fixed effects were selected a priori and included student sex, grade level (middle vs. high school), school location (central vs. peripheral), perceived disgust, enjoyment rating (where relevant), and pellet complexity (total prey items). The school’s name was included as a random intercept for student perception outcomes (enjoyment and willingness). Fixed effects in GLMMs were evaluated using F-tests as implemented in the GENLINMIXED procedure in IBM SPSS Statistics version 29.0.2 (IBM Corp., Armonk, NY, USA). School was included as a random intercept to account for non-independence among student observations. Because each participating school was typically represented by a single science teacher responsible for implementing the protocol across classes, this random effect likely captures both school-level context and variation in teacher implementation.

Collinearity among fixed effects was low (all VIF ≤ 1.47; tolerance ≥ 0.68; condition indices < 16). Residual diagnostics for linear mixed models indicated approximate normality and homoscedasticity. Model diagnostics revealed mild overdispersion (Pearson χ^2^/df = 1.58), which is within acceptable limits for binomial GLMMs and unlikely to affect inference. Accordingly, no additional correction was applied.

## 3. Results

### 3.1. Student Prey Identification Accuracy and Success

**Figure 1:**
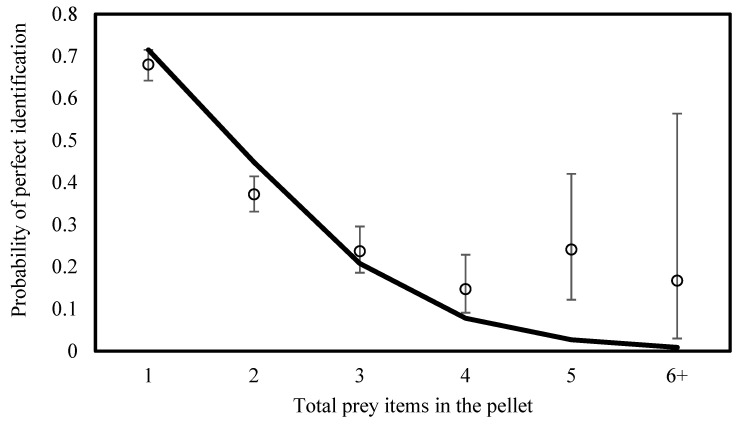
Probability of perfect prey identification (all prey items correctly identified) as a function of pellet complexity (total prey items per pellet). Points represent observed proportions (±95% CI). The solid line shows predictions from a binomial GLMM with a logit link and school as a random intercept. The “6+” category pools pellets containing ≥6 prey items due to low sample sizes (n = 628, 500, 224, 102, 29, and 6 for prey items 1–5 and 6+, respectively).

Across all pellets (N = 3333 prey items), the prey assemblage was dominated by Levant vole (*Microtus guentheri*; 49.4%, n = 1646) and house mouse (*Mus musculus*; 32.9%, n = 1098), followed by shrews (*Crocidura* spp.; 8.6%, n = 288) and Tristram’s jird (*Meriones tristrami*; 6.8%, n = 225). Less frequent prey included black rat (*Rattus rattus*; 1.2%, n = 41), birds (0.5%, n = 16), mole rat (0.03%, n = 1), other prey (0.1%, n = 4), and unrecognizable items (0.4%, n = 14). Across 1612 student–pellet attempts, 50.0% resulted in perfect identification (i.e., all prey items in the pellet were identified correctly). Across all prey items pooled across attempts, students correctly identified 62.0% of individual prey items. In 76% of student pellet attempts, students correctly identified at least one prey item. Using a binomial GLMM with a logit link and school name included as a random intercept, the probability of perfect identification decreased strongly as the number of prey items per pellet increased (β = −1.13, 95% CI −1.27 to −0.99; F_1,1610_ = 259.83, *p* < 0.001). Each additional prey item reduced the odds of perfect identification by approximately 68% (Exp(β) = 0.32, 95% CI 0.28–0.37) (Figure 1). Consistent with this effect, predicted probabilities (from the fitted model) decreased from approximately 85% for pellets containing a single prey item to ~50% for two prey items, ~30% for three prey items, and <10% for pellets containing five or more prey items, indicating a rapid loss of identification reliability as sample complexity increased. Pellets containing more prey items were, therefore, substantially more challenging for students to identify perfectly.

To determine whether identification success differed among prey taxa independent of pellet complexity, we restricted analyses to pellets containing a single prey species. Identification accuracy varied significantly among species (binomial GLMM with logit link; F_4,619_ = 13.00, *p* < 0.001). Descriptively, voles were identified correctly in 78% of cases (n = 416), shrews in 79% (n = 14), mice in 48% (n = 107), jirds in 48% (n = 75), and rats in 33% (n = 12). Using voles as the reference category, mice showed significantly higher odds of incorrect identification (β = 1.47, 95% CI 0.98–1.95; *p* < 0.001; Exp(β) = 4.33, 95% CI 2.66–7.05). Similarly, jirds had elevated odds of incorrect identification relative to voles (β = 1.44, 95% CI 0.85–2.03; *p* < 0.001; Exp(β) = 4.23, 95% CI 2.35–7.63). Rats exhibited the highest likelihood of incorrect identification (β = 2.00, 95% CI 0.70–3.30; *p* = 0.003; Exp(β) = 7.39, 95% CI 2.02–26.98). In contrast, identification accuracy for shrews did not differ significantly from voles (β = −0.25, 95% CI −1.67–1.16; *p* = 0.725). These results indicate that identification accuracy differs substantially among prey taxa even under minimal ecological complexity.

### 3.2. Teacher Participation Results

Of the 107 teachers who received kits, 71 (66.4%) returned at least one completed kit. Return rates were 74.5% (35/47) in 2018–2019 and 60.0% (36/60) in 2019–2020, but this difference was not statistically significant (χ^2^_1_ = 2.47, *p* = 0.12). Among non-returning teachers (n = 36), 22 returned kits unused and 14 did not return kits at all despite prepaid shipping. Among returners (n = 71), teachers took 1–5 kits (median = 1), and most completed all kits taken (55/71; 77.5%). The probability of returning at least one completed kit did not differ between school years (Exp(β) = 0.81, 95% CI 0.31–2.16; Wald χ^2^_1_ = 0.17, *p* = 0.678). In a logistic regression including school level, school location, and year as fixed effects, the overall model was not significant (χ^2^_3_ = 5.96, *p* = 0.114). School level showed a non-significant trend, with high-school teachers having lower odds of returning a kit compared to middle-school teachers (Exp(β) = 0.40, 95% CI 0.15–1.10; Wald χ^2^_1_ = 3.13, *p* = 0.077). School location was not associated with return probability (Exp(β) = 1.04, 95% CI 0.43–2.52; *p* = 0.931).

Among teachers who returned at least one completed kit (returners; n = 71), the mean completion rate was 84.4% (98 completed kits returned out of 116 kits taken). Using a binomial generalized linear model with a logit link (response = number of completed kits returned/total kits taken), kit completion rate was not significantly associated with school location (β = −0.45, 95% CI −1.51 to 0.62; F_1,67_ = 0.70, *p* = 0.405), school level (β = 0.31, 95% CI −0.72 to 1.34; F_1,67_ = 0.36, *p* = 0.548), or year (β = 0.65, 95% CI −0.30 to 1.60; F_1,67_ = 1.87, *p* = 0.176). The overall fixed-effects model was not significant (F_3,67_ = 0.95, *p* = 0.424). Because this analysis excludes non-returning teachers, predictors of completion rate should be interpreted as applying only to teachers who participated at least once.

Most returners also completed a brief questionnaire (59/71; 83.1%). Of those responding, 50.0% reported conducting nature-based scientific activities with students prior to this project (29/58), 20.7% had previously conducted research activities with academic researchers (12/58), and 66.1% perceived a difference between academic research and active learning research (39/59). Teachers rated all proposed project goals highly (means 4.10–4.78 on a 1–5 scale), with the highest ratings for exploring nature/environment (mean = 4.78) and developing student curiosity (mean = 4.71).

**Figure 2:**
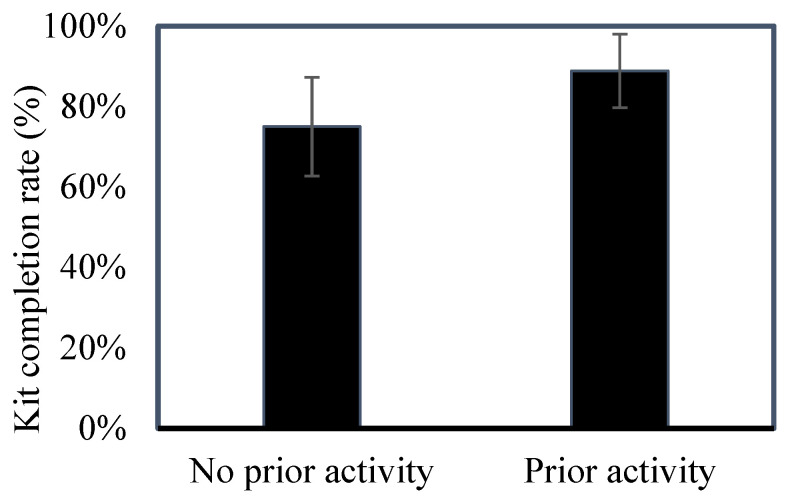
Kit completion rate (±95% CI) among returning teachers by prior classroom scientific activity. Completion rate is calculated as the number of completed kits returned divided by the number of kits taken. Teachers with prior classroom experience in science showed higher completion rates than those without.

Using a binomial generalized linear model with a logit link (response = number of completed kits returned/total kits taken), including year and teacher prior-experience variables as fixed effects, the overall model was not statistically significant (F_3,53_ = 2.08, *p* = 0.114). Completion rate did not differ by year (β = 0.53, 95% CI −0.57 to 1.62; F_1,53_ = 0.93, *p* = 0.339) or by prior classroom research collaboration with academic researchers (β = 0.40, 95% CI −0.65 to 1.46; F_1,53_ = 0.58, *p* = 0.449). However, teachers without prior classroom scientific activity had significantly lower completion rates than those with prior experience (β = −1.17, 95% CI −2.21 to −0.13; F_1,53_ = 5.12, *p* = 0.028), corresponding to approximately 69% lower odds of completing kits (Exp(β) = 0.31, 95% CI 0.11–0.88). Descriptively, teachers with prior classroom scientific activity showed higher completion rates (88.9%, 95% CI 79.7–98.1%) compared to those without prior activity (75.0%, 95% CI 62.8–87.3%) (Figure 2).

### 3.3. Student Perceptions and Engagement During the Pellet Activity

In the study, 74.9% (N = 394) of students reported that the thought of pellets was disgusting. Using a binary logistic regression GLMM with a logit link and school name included as a random intercept, students’ perception of pellets as disgusting was not significantly associated with grade level (β = −1.16, 95% CI −2.41 to 0.09, F_1,367_ = 3.33, *p* = 0.069), school location (β = 0.65, 95% CI −0.54 to 1.84, F_1,367_ = 1.15, *p* = 0.284), or gender (β = −0.10, 95% CI −0.61 to 0.41, F_1,367_ = 0.15, *p* = 0.697).

**Figure 3:**
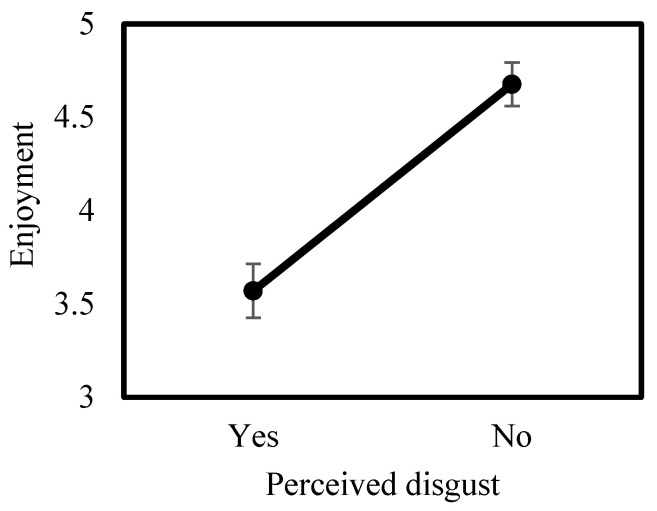
Enjoyment (1 = suffered, 5 = enjoyed a lot) of owl pellet research activity by perceived disgust (yes vs. no).

Students rated their enjoyment of the pellet activity relatively highly (mean = 3.88, SE = 0.06, N = 412; 1 = suffered, 5 = enjoyed a lot). Using a normal mixed effects model with an identity link and school name included as a random intercept, students who reported that pellets were disgusting had lower enjoyment scores than students who did not report disgust (β = −1.06, 95% CI −1.32 to −0.79, F_1,362_ = 62.72, *p* < 0.001; Figure 3). Enjoyment was not significantly related to grade level (β = 0.35, 95% CI −0.24 to 0.94, F_1,362_ = 1.36, *p* = 0.25), school location (β = −0.13, 95% CI −0.67 to 0.41, F_1,362_ = 0.22, *p* = 0.64), or gender (β = −0.18, 95% CI −0.41 to 0.06, F_1,362_ = 2.25, *p* = 0.14).

**Figure 4:**
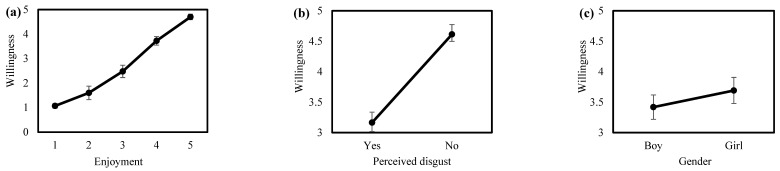
Willingness (1 = no, 5 = definitely) to analyze owl pellets again as a function of (**a**) enjoyment (1 = suffered, 5 = enjoyed a lot) of the pellet activity, (**b**) by perceived disgust, and (**c**) by gender.

When asked whether they would want to analyze pellets again, students reported moderate to high willingness (mean = 3.56, SE = 0.07, N = 408; 1 = no, 5 = definitely). Using a normal mixed effects model with an identity link and school name included as a random intercept, willingness to repeat the activity differed strongly by enjoyment rating (F_4,358_ = 123.11, *p* < 0.001). Students who reported lower enjoyment (scores 1–4) were less willing to participate again than those who reported the highest enjoyment (score 5). For example, students who rated enjoyment as 1 had substantially lower willingness than those rating enjoyment as 5 (β = −3.33, 95% CI −3.70 to −2.96; *p* < 0.001; Figure 4a). Students who perceived pellets as disgusting were also less willing to repeat the activity than those who did not report disgust (β = −0.44, 95% CI −0.67 to −0.22, F_1,358_ = 15.56, *p* < 0.001; Figure 4b). In addition, boys reported slightly lower willingness than girls (β = −0.23, 95% CI −0.41 to −0.05, F_1,358_ = 6.34, *p* = 0.012; Figure 4c). Willingness was not related to grade level (β = 0.11, 95% CI −0.19 to 0.41, F_1,358_ = 0.53, *p* = 0.47) or school location (β = 0.03, 95% CI −0.25 to 0.30, F_1,358_ = 0.04, *p* = 0.85).

Educational level (middle vs. high school) did not significantly influence student identification accuracy, disgust, enjoyment, or willingness to repeat the activity, indicating that the complexity-driven effects observed were consistent across age groups.

## 4. Discussion

### 4.1. Identification Accuracy and Limits to Ecological Inference

Student prey-identification accuracy was moderate. Across all prey items, 62% were correctly identified, and half of the student pellet attempts resulted in perfect identification. Most students correctly identified at least one prey item per pellet, indicating partial task mastery. However, ecological inference, especially regarding species-level diet composition, proportional abundance estimation, and rare-species detection, demands higher reliability than just partial success [11]. Similar patterns of training-dependent identification accuracy have been documented in other citizen science contexts, where volunteer performance improves with structured training but remains sensitive to task difficulty [47,50].

International evaluations of volunteer-based biodiversity monitoring in Europe and North America have similarly shown that identification accuracy declines with increasing task difficulty and taxonomic complexity, even under structured training protocols [19,37,47]. Together, these international findings indicate that complexity-driven identification error is a recurrent structural constraint in citizen science systems, rather than a context-specific limitation of the present study.

A systematic review of volunteer-based biological surveys further demonstrated that data quality is influenced by both volunteer characteristics and project design features, including prior knowledge, sampling structure, and training protocols [37,51]. Notably, comparisons between volunteers and professionals do not consistently show superior professional performance when evaluated against common shared reference standards [19,47], suggesting that variability in data quality reflects structural and contextual factors rather than categorical differences between participant types. These findings reinforce the importance of explicitly identifying the conditions under which citizen-generated data remain reliable.

Even in a relatively simple prey community dominated by two rodent taxa, overall identification accuracy was not high enough to assume reliable species-level estimates without expert verification. At this level of error, misidentifications may distort proportional diet estimates, suppress or inflate apparent species richness, and obscure rare taxa [27,37]. While coarse grouping of dominant prey categories may remain broadly informative, fine-resolution ecological conclusions derived solely from student identifications would be vulnerable to bias. Whole-pellet identification reliability declined systematically with pellet complexity.

Ecological complexity, operationalized here as the number of prey remains in a pellet, was the strongest predictor of identification accuracy. As pellet complexity increased, the probability of perfect identification declined sharply, indicating that the structural properties of biological samples impose measurable constraints on student performance. This pattern has direct implications for biodiversity research: when identification tasks involve multiple morphologically similar taxa or complex community compositions, citizen-generated data may underestimate species richness or misclassify rare species unless validation mechanisms are in place [19,52]. Thus, ecological complexity should be treated as a design variable in school-based monitoring programs rather than assumed to be a neutral background condition.

Notably, educational level did not significantly modify this pattern. Middle and high school students exhibited similar declines in identification accuracy with increasing pellet complexity, suggesting that structural task demands rather than developmental differences drive the observed performance constraints. This pattern shows that data quality in school-based citizen science depends not only on observer training but also on the interaction between human participants and the ecological complexity of what they interpret.

### 4.2. Implementation Feasibility in School-Based Monitoring

This study shows that school-based participation can generate substantial sampling effort for ecological monitoring in real classroom settings. About two-thirds of teachers who received kits returned at least one complete set of materials, and most teachers who returned kits completed all the kits they took. These participation rates are encouraging, given that implementation occurred without researcher supervision and within existing curriculum constraints. However, one-third of teachers did not return usable materials, indicating that non-participation remains an important practical limitation when scaling school-based monitoring. Return probability did not differ significantly by year, school level, or school location in the multivariable model, although there was a non-significant tendency for high school teachers to be less likely than middle school teachers to return a completed kit. Among returning teachers, kit completion rate was also not significantly associated with year, school level, or school location. These results indicate that implementation was broadly feasible across school contexts, although teacher follow-through was variable [53,54].

Among the subset of returning teachers who completed the background questionnaire, prior collaboration with academic researchers was not associated with completion rate, whereas teachers without prior experience conducting scientific activities in the classroom had lower completion rates than those with such experience. This suggests that successful implementation may depend less on formal ties to academic research and more on teachers’ familiarity and confidence with classroom-based scientific activities [40,55,56]. For future school-based monitoring programs, support should focus not only on the scientific protocol but also on enhancing teachers’ practical ability to conduct hands-on inquiry in classrooms [40,55,56].

Despite initial disgust responses, enjoyment remained high, and willingness to repeat the activity was driven mainly by positive experiences. Citizen science projects can improve participants’ understanding of science and increase engagement when carefully designed to include authentic research activities [57,58]. Affective responses, therefore, did not stop large-scale participation. From a monitoring standpoint, these results show that schools can be effective partners in expanding sample-processing capacity, but participation dropout needs to be planned for when designing large-scale sampling networks.

To improve data quality while preserving educational value, task complexity should be aligned with students’ experience, starting with simpler samples and progressing to more diverse pellets. A structured validation mechanism, such as peer cross-checking or expert review of high-complexity samples, can further enhance reliability [37,43]. In addition, explicit instructional scaffolding and standardized identification protocols may improve consistency in classroom implementation [40,55]. Together, these strategies enable school-based citizen science to balance authentic inquiry with robust biodiversity monitoring.

### 4.3. Task Complexity as a Structural Constraint on Citizen-Science Data Quality

The probability of perfect identification declined sharply as pellet complexity increased, showing that whole-pellet identification reliability decreases as task complexity increases. Increased training and standardized protocols did not fully overcome these limitations, and similar patterns of persistent misclassification have been reported in other citizen science studies [50,59]. Consistent with our findings, pellets containing multiple prey items, common in productive agricultural systems [6], are more likely to result in incomplete or incorrect identifications when processed by non-specialists, particularly as task complexity increases [59]. As prey richness increases, estimates of diet diversity based on student data may become increasingly unreliable. Rare species may be particularly vulnerable because even a few misclassifications can result in non-detection [52]. Monitoring programs relying solely on unverified student identifications may therefore underestimate diversity or misrepresent species’ relative abundance in more complex samples [60].

Species-level variation in identification success demonstrates that observer error is not uniform across taxa but reflects structured interactions between morphological distinctiveness and observer expertise [61]. Such non-random error patterns have direct implications for biodiversity inference: taxa that are morphologically similar may be systematically under-detected or misclassified, potentially distorting estimates of relative abundance and community composition [18]. Incorporating species-specific difficulty into validation protocols may therefore improve the interpretability of student-generated ecological datasets without diminishing the educational value of participation [43,47].

### 4.4. Limitations and Future Directions

Several limitations should be acknowledged. First, identification accuracy was assessed in classroom settings without direct researcher supervision, and differences among teachers in instructional emphasis may have influenced performance variations that our models did not fully account for [62]. Second, I measured overall accuracy but did not differentiate error types (e.g., systematic misclassification among specific taxa), which could help improve estimates of ecological bias [63]. Third, student-level independence in classrooms might not have been fully upheld, as peer discussion during identification could have influenced results. Finally, our conclusions are based on a prey community dominated by a small number of rodent taxa; identification performance might vary in regions with higher taxonomic diversity or more morphologically similar species, where volunteer-based identification has been shown to miss a substantial proportion of taxa [64].

One potential avenue for improving reliability while maintaining broad participation is the development of an AI-based smartphone application capable of automated species-level identification of skeletal items. Automated species recognition systems are already widely used in ecological research, including deep-learning approaches for camera-trap image classification and plant identification [65,66,67], demonstrating the feasibility of computer-vision applications in biodiversity monitoring. However, any such system would require training on large expert-labeled datasets and independent validation against expert evaluations before it could be used reliably in school-based monitoring [68]. When combined with structured validation frameworks and school-based participation, this approach can both expand monitoring capacity and offer meaningful scientific engagement for students [38,69]. Since owl pellets are already distributed commercially to schools for anatomical study [70], integrating verified geospatial metadata into these supply chains could turn an existing educational product into a distributed ecological monitoring resource. Georeferenced pellet sourcing would facilitate repeated sampling across regions and involve students in authentic data collection.

Future research should focus on quantifying taxon-specific misclassification patterns [50], assessing the performance of structured digital identification tools [67], and testing tiered validation frameworks that combine wide educational participation with targeted expert oversight [37]. Such efforts would help determine the conditions under which school-based pellet analysis can reliably contribute to long-term ecological monitoring.

These findings highlight several methodological priorities for future research. Because pellet complexity was not experimentally controlled but naturally varied across samples, stratified sampling or controlled-complexity treatments would enable more precise isolation of causal effects. Although teacher participation and prior experience were evaluated, classroom-level instructional mediation was not directly measured; variation in scaffolding strategies, time allocation, or pedagogical approach may influence identification accuracy independently of ecological complexity. Incorporating structured observation or standardized facilitation protocols would clarify how teacher mediation moderates complexity-driven error. Finally, because student engagement measures were based on self-report questionnaires, integrating performance-based or longitudinal assessments would further strengthen inference about the relationship between affect and sustained participation. Addressing these methodological dimensions will enhance the reliability and scalability of school-based citizen science monitoring frameworks.

## 5. Conclusions

This study demonstrates that ecological complexity systematically constrains student identification accuracy in school-based citizen science programs. Although student engagement and teacher participation were strong, identification reliability declined sharply as pellet complexity increased.

For educational applications, these findings suggest that instructors should align task difficulty with students’ experience and provide additional scaffolding when working with biologically complex samples. Structured validation steps, such as expert review of high-complexity samples or peer verification protocols, may help maintain both educational value and data reliability.

For conservation and biodiversity monitoring, the results indicate that student-generated data can contribute meaningfully to large-scale sampling efforts when complexity is moderate and validation procedures are in place. However, programs aiming to detect rare species or conduct species-level inference under high ecological complexity should incorporate formal quality-control mechanisms, including stratified sampling, expert confirmation, or AI-assisted identification tools.

By explicitly accounting for ecological complexity in project design, school-based citizen science programs can balance educational engagement with reliable ecological monitoring outcomes.

## Data Availability

The data presented in this study are not publicly available due to privacy and ethical restrictions associated with anonymous questionnaire data collected from students in a school-based setting.
